# Current Position of Nuclear Medicine Imaging in Primary Bone Tumors

**DOI:** 10.3390/diagnostics15212786

**Published:** 2025-11-03

**Authors:** Narae Lee, Min Wook Joo

**Affiliations:** 1 Division of Nuclear Medicine, Department of Radiology, St. Vincent’s Hospital, College of Medicine, The Catholic University of Korea, 222 Banpo-daero, Seocho-gu, Seoul 06591, Republic of Korea; lnr84@catholic.ac.kr; 2 Department of Orthopaedic Surgery, St. Vincent’s Hospital, College of Medicine, The Catholic University of Korea, 222 Banpo-daero, Seocho-gu, Seoul 06591, Republic of Korea

**Keywords:** bone tumor, nuclear medicine, bone scan, SPECT/CT, [^18^F]FDG

## Abstract

Primary bone tumors encompass a heterogeneous spectrum ranging from benign entities to highly aggressive sarcomas. This review aims to summarize the current role and future perspectives of nuclear medicine in the diagnosis, staging, and management of primary bone tumors. Accurate diagnosis and staging are critical yet challenging due to histologic heterogeneity and overlapping imaging features. While radiographs, computed tomography (CT), and magnetic resonance imaging (MRI) remain essential, nuclear medicine provides a complementary functional perspective by assessing bone turnover, vascularity, and glucose metabolism. Bone scintigraphy is highly sensitive for skeletal lesions and useful for detecting skip lesions or multifocal disease, although its specificity is limited. Hybrid single-photon emission computed tomography (SPECT)/CT enhances diagnostic confidence through precise anatomic localization and quantitation. [^18^F]fluorodeoxyglucose ([^18^F]FDG) positron emission tomography (PET)/CT, by directly visualizing tumor metabolism, has become a cornerstone in osteosarcoma and Ewing sarcoma management, demonstrating superiority over bone scintigraphy for detecting skeletal metastases. In chondrosarcoma, [^18^F]FDG uptake correlates with histologic grade, although overlap with benign cartilage tumors complicates interpretation. Future directions include the integration of quantitative SPECT, artificial intelligence, and novel tracers such as [^18^F]sodium fluoride and [^68^Ga]Ga-fibroblast activation protein inhibitor (FAPI). Collectively, nuclear medicine imaging is becoming a key element in musculoskeletal oncology, offering unique biological insights that complement anatomic imaging and contribute to improved patient management.

## 1. Introduction

### 1.1. The Diagnostic Challenge of Primary Bone Tumors

Primary bone tumors represent a heterogeneous group of neoplasms, encompassing a wide range of histologic subtypes from indolent benign lesions to highly aggressive sarcomas [1,2,3]. While certain lesions such as osteochondromas are relatively common, others—including high-grade sarcomas—are exceedingly rare [4]. As both imaging interpretation and biopsy of these lesions are complex, expert coordination among radiologists, pathologists, orthopedic oncologists, and other specialists is essential.

### 1.2. The Unique Contribution of Nuclear Medicine: A Functional Perspective

Whereas conventional radiological modalities such as X-ray, computed tomography (CT), and magnetic resonance imaging (MRI) depict detailed anatomy, nuclear medicine provides a fundamentally different yet complementary functional perspective of bone tumors [2]. For example, nuclear medicine techniques can assess regional blood flow, bone turnover, cellular glucose metabolism, or receptor expression, thereby characterizing a tumor’s biology across the entire body in a single exam. This whole-body functional assessment is critically important at multiple stages of care. In the initial workup of a suspected bone malignancy, nuclear imaging plays a pivotal role for systemic staging, detecting distant skeletal or soft tissue metastases that might be missed by localized imaging. In patients with large or heterogeneous tumors, functional imaging can guide biopsy to the most metabolically active (and likely highest-grade) region, improving diagnostic yield by reducing sampling error [5]. Furthermore, by quantifying tumor metabolism before and after therapy, nuclear medicine enables the early assessment of treatment response [6,7]. Therefore, modern bone tumor diagnosis relies on a synergistic integration of anatomic and functional imaging. Nuclear medicine does not replace CT or MRI for local tumor delineation. Rather, this synergy addresses key clinical questions regarding tumor metabolic activity, whole-body disease burden, and therapeutic efficacy, ultimately improving diagnostic accuracy and patient management.

### 1.3. Objectives and Scope of This Review

Recent reviews have examined positron emission tomography (PET)-based modalities in musculoskeletal oncology. Hassan et al. summarized the available literature on [^18^F] fluorodeoxyglucose ([^18^F]FDG) PET/CT imaging’s utility in the management of primary bone tumors including osteosarcoma, chondrosarcoma, and Ewing sarcoma [6]. Likewise, Heras et al. emphasized the advantage of PET/MRI in delineating tumor margins and assessing treatment response [7]. However, PET/MRI remains limited by its lower global availability compared with PET/CT and by suboptimal detection of small pulmonary metastases, for which CT remains preferred modality.

Building upon these prior works, the present review expands the discussion beyond PET-based imaging to include single-photon emission tomography (SPECT)/CT, quantitative and radiomics-driven analysis, and the emerging role of artificial intelligence—thereby offering a broader nuclear medicine perspective encompassing both benign and malignant primary bone tumors.

## 2. Nuclear Medicine Modalities

### 2.1. Bone Scintigraphy and SPECT/CT

Bone scintigraphy, often referred to as a bone scan, uses an intravenously administered Technetium-99m ([^99m^Tc])-labeled diphosphonate (e.g., [^99m^Tc]Tc-methylene diphosphonate, MDP) that adsorbs onto hydroxyapatite in bone, reflecting osteoblastic activity and regional blood flow. Although sensitivity data for primary bone tumors are limited and vary depending on tumor type, skeletal site, and methodology, bone scintigraphy is generally considered highly sensitive for skeletal lesions—changes as small as 5% in bone formation can be detected, whereas 40–50% mineral loss is required before changes on radiographs become evident [8]. Moreover, bone scintigraphy remains valuable for identifying multifocal bone lesions, skip lesions, or distant skeletal metastases within a single whole-body examination.

However, planar scintigraphy provides limited spatial resolution and specificity, as increased tracer uptake may occur in numerous benign or physiological conditions, including fractures, degenerative changes, infection, and inflammation. Physiological uptake can also be seen at the costochondral junctions, sacroiliac joints, and open growth plates, potentially mimicking neoplastic lesions [9]. These pitfalls highlight the importance of correlating scintigraphic findings with anatomical imaging and clinical context. A three-phase bone scan (perfusion, blood-pool, and delayed imaging) can improve specificity by distinguishing vascularity and bone turnover patterns [10]. The integration of SPECT enhances lesion detection by providing three-dimensional imaging that removes overlapping activity and improves contrast resolution. Hybrid SPECT/CT further fuses functional data from scintigraphy with precise anatomic localization from CT, substantially improving diagnostic accuracy and reader confidence [11,12,13]. As a result, SPECT/CT has become an increasingly utilized and important adjunct for the functional evaluation of suspected bone tumors in current clinical practice.

### 2.2. [^18^F]FDG PET/CT

The advent of [^18^F]FDG PET/CT represents a shift from assessing bone reactions to evaluating tumor biology directly. [^18^F]FDG, a glucose analog, is transported into cells and phosphorylated, becoming metabolically trapped in proportion to glycolytic activity. The intensity of uptake, typically expressed as the standardized uptake value (SUV), correlates with tissue metabolic rate, and malignant tumors generally demonstrate higher values than benign processes [14]. The maximum standardized uptake value (SUVmax)—defined as the highest voxel value of [^18^F]FDG uptake within a lesion, normalized to injected dose and body weight—is the most widely used semiquantitative parameter for assessing tumor metabolism and treatment response in bone sarcomas [6,15].

PET is now performed almost exclusively with hybrid PET/CT scanners, enabling precise anatomical localization of [^18^F]FDG-avid lesions. Compared with bone scintigraphy, [^18^F]FDG PET/CT provides superior sensitivity for lytic or marrow-based metastases and simultaneously identifying osseous and extra-osseous disease in a single whole-body study. It is also less affected by artifacts from metallic implants [16]. These advantages have established [^18^F]FDG PET/CT as a key modality for staging, restaging, and monitoring therapy in bone sarcomas [17,18,19,20,21,22,23,24,25,26,27].

The primary limitation of [^18^F]FDG PET/CT is its lack of specificity: the uptake of [^18^F]FDG occurs not only in malignant tumors but also in inflammatory or infectious sites and in some benign bone tumors, which may show intense uptake indistinguishable from malignancy. Therefore, interpretation requires careful correlation with clinical context and morphological imaging [16,28].

## 3. The Role of Nuclear Medicine in Primary Malignant Bone Tumors

### 3.1. Osteosarcoma

Osteosarcoma is the most common primary malignant bone tumor, with a peak incidence in adolescents aged 10–14 years and a second peak in adults older than 60 years. It typically affects metaphysis of long bone in adolescents and young adults, and its management relies heavily on accurate staging and assessment of response to chemotherapy [29,30].

#### 3.1.1. Diagnosis and Staging

Osteosarcomas are generally highly metabolic tumors and are therefore intensely [^18^F]FDG-avid on PET/CT [21,31]. This property makes [^18^F]FDG PET/CT a cornerstone of the initial staging workup, providing a single whole-body examination to detect osseous, pulmonary, and other soft-tissue metastases. Multiple studies demonstrated the superiority of [^18^F]FDG PET/CT over conventional bone scintigraphy for the detection of skeletal metastases in osteosarcoma [17,27,32,33,34]. This advantage is particularly evident for lytic metastases or those located near the active growth plates, where physiologic uptake on bone scintigraphy can mask adjacent lesions.

#### 3.1.2. Biopsy Guidance and Grading

Osteosarcomas are often large, heterogeneous tumors containing areas of variable histologic grade, necrosis, and hemorrhage. This heterogeneity poses a significant challenge for biopsy, which is susceptible to sampling error that can lead to an underestimation of the tumor’s histologic grade. [^18^F]FDG PET/CT provides a unique solution to this problem. A metabolic map generated by [^18^F]FDG PET/CT enables identification of the most metabolically active and typically highest-grade components of the neoplasm [35,36]. Using PET/CT to guide the biopsy leads to more accurate histologic grading and appropriate treatment planning.

#### 3.1.3. Treatment Response Assessment

**Figure 1:**
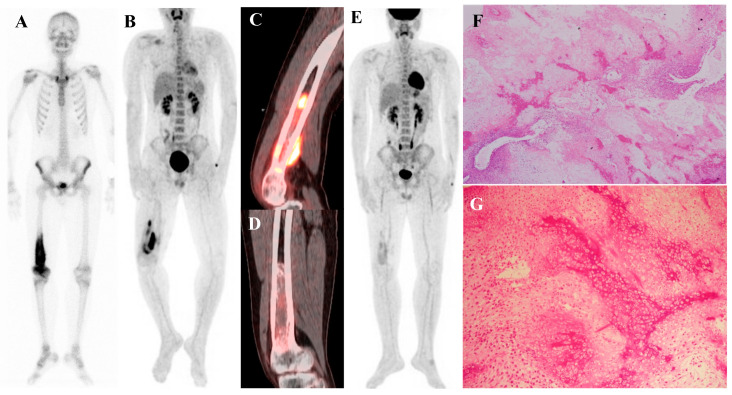
A 16-year-old male with osteosarcoma of the right distal femur. (**A**) Pretreatment bone scintigraphy shows intense uptake in the distal femoral metaphysis. (**B**,**C**) Pretreatment [^18^F]FDG positron emission tomography/computed tomography demonstrates a heterogeneous, [^18^F]FDG-avid mass with cortical breakthrough and soft-tissue extension (SUVmax 12.6). (**D**,**E**) After neoadjuvant chemotherapy, [^18^F]FDG uptake markedly decreased (SUVmax 2.3) with reduction in tumor extent. (**F**) Wide excision specimen (×40) revealed predominantly necrotic tissue, confirming a marked response to chemotherapy, in contrast to (**G**) the pretreatment biopsy specimen (×100) with viable malignant cells. He has remained disease-free for 8 years.

Perhaps the most critical role for [^18^F]FDG PET/CT in osteosarcoma management is in the evaluation of response to neoadjuvant chemotherapy. The standard of care for high-grade osteosarcoma involves preoperative chemotherapy followed by surgical resection. The degree of tumor necrosis in the resected specimen is one of the most powerful prognostic factors. A “good response,” defined as ≥90% tumor necrosis, is associated with a much better prognosis than a “poor response” (<90% necrosis). [^18^F]FDG PET/CT perfor-med before and after neoadjuvant chemotherapy can non-invasively predict this histologic response. A significant reduction in the tumor’s SUVmax following chemotherapy is a robust indicator of a good pathologic response [15,37,38,39,40,41,42,43]. This information can help predict prognosis and guide critical therapeutic decisions. Figure 1 demonstrates a representative case in which marked reduction of [^18^F]FDG uptake after chemotherapy corresponded to predominantly necrotic tissue in the resected specimen, resulting in long-term disease-free survival. This information can help predict prognosis and guide critical therapeutic decisions.

#### 3.1.4. Detection of Recurrence

Osteosarcoma possesses the potential to metastasize to various organs. According to a meta-analysis evaluating the diagnostic utility of [^18^F]FDG PET/CT for detecting recurrence following treatment completion, this imaging modality demonstrates excellent performance. The pooled data from seven studies yielded a composite sensitivity of 91% (95% CI, 81% to 96%) and a composite specificity of 93% (95% CI, 87% to 97%) [44]. These high-performance metrics establish [^18^F]FDG PET/CT as a reliable and crucial tool for the post-treatment surveillance of osteosarcoma patients. This level of accuracy is critical for making timely and appropriate decisions regarding further clinical management.

### 3.2. Ewing Sarcoma

Ewing sarcoma is an aggressive small round blue cell tumor of bone and soft tissue that primarily affects children and young adults. It most commonly arises in the pelvis, femur, humerus, ribs and spine. It has a high propensity for early metastasis, most commonly to the lungs and other bones, making accurate initial staging essential for determining prognosis and treatment [45,46].

#### 3.2.1. Diagnosis and Staging

Like osteosarcoma, Ewing sarcoma is typically a highly [^18^F]FDG-avid malignancy [21,31]. However, the superiority of [^18^F]FDG PET/CT over bone scintigraphy for staging is even more pronounced in Ewing sarcoma [19,20]. The reason lies in the tumor’s biology: Ewing sarcoma metastases are often purely lytic and reside within the bone marrow, provoking little to no reactive new bone formation. As bone scintigraphy relies on detecting this osteoblastic reaction, it can easily miss these lesions. In contrast, [^18^F]FDG PET/CT directly images the metabolically active tumor cells within the marrow. Consequently, studies have demonstrated a dramatic difference in sensitivity. One study reported a sensitivity of 88% for PET/CT in detecting bone metastases from Ewing sarcoma, compared to just 37% for conventional imaging, which included bone scan [47]. Another study found sensitivities of 88% for PET/CT versus 50% for bone scan [22]. Given this clear evidence, [^18^F]FDG PET/CT is now considered the preferred nuclear medicine modality for the systemic staging of Ewing sarcoma.

#### 3.2.2. Detection of Recurrence

**Figure 2:**
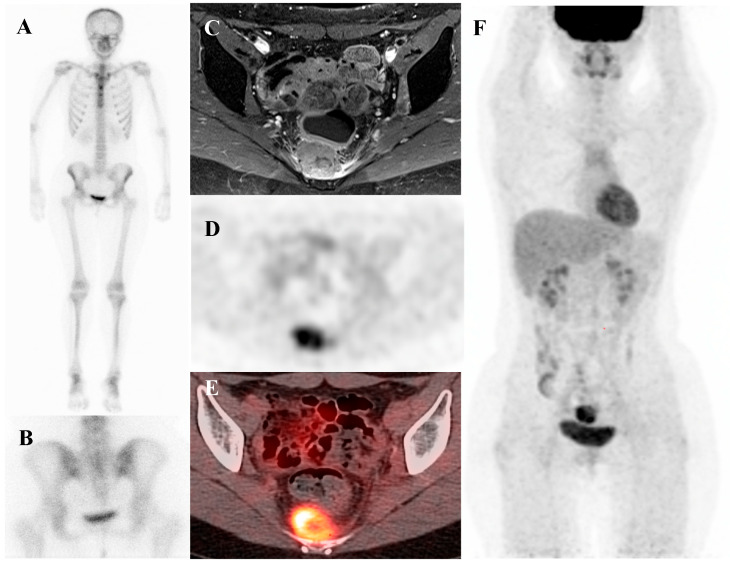
A 19-year-old female with recurrent Ewing’s sarcoma. (**A**,**B**) Bone scintigraphy shows no abnormal uptake on both anterior and posterior view. (**C**) T1 fat-suppressed magnetic resonance imaging demonstrates a lobulated, enhancing presacral mass with invasion of the sacrum. (**D**–**F**) [^18^F]FDG positron emission tomography/computed tomography reveals intense uptake of the mass (SUVmax 9.0). Surgical excision confirmed recurrence of Ewing’s sarcoma.

Patients with Ewing sarcoma are at high risk for disease recurrence, which most often occurs in the lungs or bone and carries a poor prognosis [45,46]. [^18^F]FDG PET/CT is an extremely valuable tool for post-treatment surveillance and for the early detection of recurrent disease [18,25]. The appearance of new, focal [^18^F]FDG-avid lesions on follow-up scans is highly suspicious for recurrence and prompts further investigation and salvage therapy. Whole-body nature of [^18^F]FDG PET/CT is ideal for detecting recurrence at any potential site. Figure 2 demonstrates a representative case in which bone scintigraphy failed to reveal recurrent disease, whereas [^18^F]FDG PET/CT clearly identified the metabolically active presacral mass, subsequently confirmed as recurrent Ewing sarcoma after surgical excision.

### 3.3. Chondrosarcoma

Chondrosarcoma is a malignant tumor arising from cartilage-producing cells and represents the second most common primary bone sarcoma after osteosarcoma, typically occurring in adults [48]. Primary chondrosarcomas can arise from normal bone, most commonly long bones and pelvis, or secondarily after malignant transformation of a preexisting enchondroma or osteochondroma [48,49,50]. It presents a different set of diagnostic challenges compared to osteosarcoma and Ewing sarcoma [24]. While high-grade chondrosarcomas can be aggressive, many are low-grade and slow-growing. The most critical clinical and imaging challenge lies not in detecting early metastases, but in accurately distinguishing enchondromas and atypical cartilaginous tumors (ACT; grade 1 chondrosarcoma of the appendicular skeleton) from more aggressive chondrosarcomas, and higher-grade chondrosarcomas [23,26]. This distinction is paramount because it dictates management. Benign enchondromas and many ACTs can be observed or treated with simple curettage, due to their limited metastatic potential, whereas high grade tumors require wide surgical resection [50,51,52].

#### 3.3.1. [^18^F]FDG PET/CT

While biopsy is the traditional gold standard for diagnosis, it is particularly problematic in cartilaginous tumors. These tumors are often large and histologically heterogeneous, and a needle biopsy may sample a low-grade area while missing a focus of high-grade disease elsewhere in the lesion. This leads to a high rate of sampling error and potential under-grading of the tumor [53].

[^18^F]FDG PET/CT has emerged as a powerful, non-invasive tool to address this diagnostic dilemma. There is a strong and well-established correlation between the metabolic activity of a cartilaginous tumor, as measured by its SUVmax on [^18^F]FDG PET, and its histologic grade [26]. The underlying principle is that higher-grade, more aggressive tumors have a higher metabolic rate and thus higher glucose uptake. Benign enchondromas, ACTs and grade 1 chondrosaromas are typically metabolically indolent and demonstrate low [^18^F]FDG uptake. In contrast, grade 2 and grade 3 chondrosarcomas are more metabolically active and show significantly higher [^18^F]FDG uptake [54,55]. Multiple studies have proposed different SUVmax cutoff values for differentiating benign from malignant lesions as well as for grading chondrosarcomas. However, no universally accepted threshold has been established.

#### 3.3.2. Bone SPECT/CT

Although [^18^F]FDG PET/CT plays a pivotal role for metabolic assessment, not all institutions have PET readily available, and many cartilaginous tumors demonstrate relatively low [^18^F]FDG uptake. In this context, bone SPECT/CT offers an alternative functional imaging technique with emerging quantitative capabilities. Historically, bone scintigraphy was explored to distinguish enchondromas from chondrosarcomas. Chondrosarcomas often show heterogeneously intense radionuclide uptake (often exceeding that of normal bone, e.g., anterior iliac crest), whereas enchondromas usually have little to no abnormal uptake [56]. Conventional SPECT alone is qualitative, but modern SPECT/CT allows semi-quantitative measurement of uptake analogous to PET. In one study conducted in 2020, ACTs had significantly higher SPECT-derived tumor SUVmean and SUVmax than enchondromas, yielding ~86% sensitivity and 75% specificity for differentiation. This demonstrates that SPECT/CT can detect the increased osteoblastic activity associated with malignancy in cartilage lesions [57].

Also, this functional heterogeneity on SPECT can be clinically exploited. A focal region of markedly increased uptake on bone SPECT/CT likely corresponds to a higher-grade focus within a cartilaginous tumor. Indeed, a recent report demonstrated that the most radiotracer-avid region of a chondrosarcoma corresponded to the high-grade histology on pathology, suggesting that bone SPECT/CT aid in selecting the optimal biopsy site for diagnosis [58].

**Figure 3:**
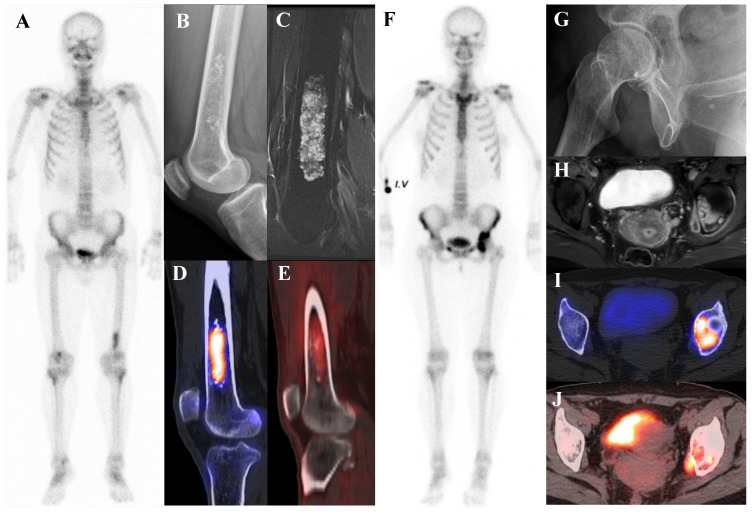
(**A**–**E**) A 54-year-old male with atypical cartilaginous tumor (ACT) of the left distal femur. (**A**) Bone scintigraphy shows increased uptake in the distal metaphysis. (**B**) Radiograph demonstrates stippled calcifications suggestive of a chondroid tumor. (**C**) T2-weighted fat-suppressed magnetic resonance imaging (MRI) shows a lobulated intramedullary lesion with heterogeneous signal and equivocal cortical erosion. (**D**) Bone single-photon emission tomography (SPECT)/computed tomography (CT) demonstrates focal uptake in the distal femur, corresponding to a calcified chondroid mass. (**E**) [^18^F]FDG positron emission tomography (PET)/CT shows mild, elongated uptake (SUVmax 2.96) in the lesion with characteristic rings-and-arcs calcification on CT. Surgical excision confirmed ACT. (**F**–**J**) A 49-year-old female with high-grade chondrosarcoma of the left acetabulum (**F**) Bone scintigraphy shows intense uptake. (**G**) Radiograph demonstrates a geographic radiolucent lesion. (**H**) T1-weighted fat-suppressed MRI reveals an infiltrative lesion with extraosseous and intra-articular extension, heterogeneous enhancement. (**I**) SPECT/CT reveals increased uptake in the acetabular region, corresponding to the infiltrative lesion with extraosseous extension. (**J**) [^18^F]FDG PET/CT demonstrates intense localized uptake in the left acetabulum with extension into the obturator internus muscle, especially pronounced at the site of extraosseous spread (SUVmax 8.4). Initial biopsy suggested grade I chondrosarcoma; however, wide excision confirmed dedifferentiated chondrosarcoma.

Figure 3 illustrates the spectrum of cartilaginous tumors, from low-grade ACT with mild metabolic activity to high-grade chondrosarcoma with aggressive infiltration and intense uptake, emphasizing the importance of integrating multimodal imaging with histopathology for accurate diagnosis and grading.

## 4. Nuclear Medicine Findings in Benign Bone Tumors and Tumor-like Lesions

A significant challenge in musculoskeletal tumor imaging is the differentiation of benign from malignant lesions. While [^18^F]FDG PET/CT is a cornerstone for staging known malignancies, its utility in initial diagnosis is complicated by the fact that many benign entities can be metabolically active and mimic cancer. This underscores a critical principle: the interpretation of nuclear medicine studies should not be interpreted in isolation. The absolute intensity of tracer uptake (e.g., SUVmax) is often less important than the pattern of uptake, the clinical context (patient age, symptoms), and careful correlation with anatomic imaging findings (lesion location, radiographic features). Nuclear medicine findings in benign tumors span the entire spectrum from photopenic (“cold”) to intensely hypermetabolic, proving that high uptake alone is not a reliable indicator of malignancy.

### 4.1. Bone-Forming Lesions

#### 4.1.1. Osteoid Osteoma

**Figure 4:**
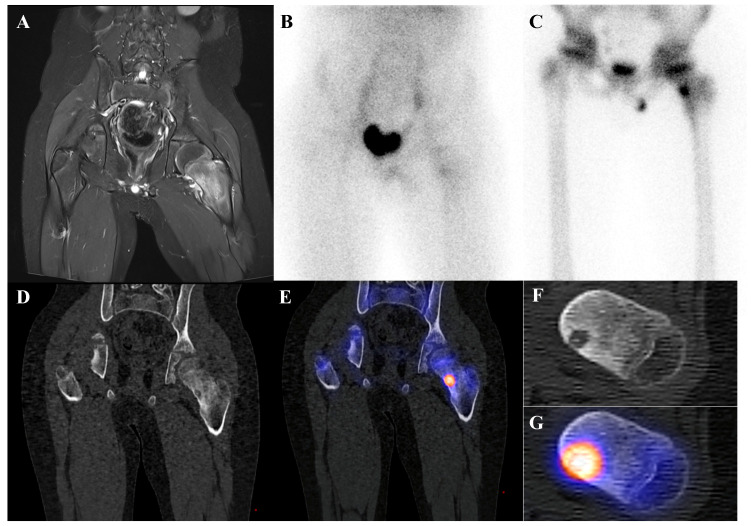
An 11-year-old male with osteoid osteoma of the left femoral neck. (**A**) T2-weighted fat-suppressed magnetic resonance imaging shows a lesion with high signal intensity and a sclerotic rim in the medial cortex, accompanied by perilesional edema (“half-moon sign”). (**B**,**C**) Bone scintigraphy demonstrates increased uptake in the left femoral neck on both blood pool and delayed phases. (**D**–**G**) Bone single-photon emission tomography/computed tomography reveals an osteolytic lesion with a central nidus and intense uptake.

Osteoid osteoma is a small, benign bone-forming tumor that classically presents in young males with nocturnal pain relieved by nonsteroidal anti-inflammatory drugs. It has a pathognomonic appearance on a three-phase bone scan. Due to its highly vascular nidus and intense osteoblastic activity, the lesion demonstrates increased perfusion on flow images, hyperemia on blood pool images, and intense, focal uptake on delayed images [59]. This intense focal uptake on the delayed phase is often referred to as the “double-density sign,” characterized by a central hot spot of extreme avidity (the nidus) surrounded by a less intense but still avid zone of reactive sclerosis [60]. Bone scintigraphy has a sensitivity approaching 100% for detecting osteoid osteomas [61]. Although benign lesions may also demonstrate increased tracer uptake, reducing the specificity of this technique, the integration of SPECT/CT substantially enhances diagnostic accuracy by providing precise anatomic localization and improved lesion characterization [62]. It is particularly valuable for localizing small or intra-articular lesions that may be occult or difficult to identify on plain radiographs. While [^18^F]FDG PET/CT usually shows high metabolic activity in the nidus [63], some osteoid osteomas demonstrate no uptake [64]. Despite this variability, [^18^F]FDG PET/CT can be useful for assessing treatment response [65]. Figure 4 shows an osteoid osteoma of the femoral neck, where bone scintigraphy and SPECT/CT clearly identified the nidus with intense uptake in concordance with MRI findings.

#### 4.1.2. Osteoblastoma

**Figure 5:**
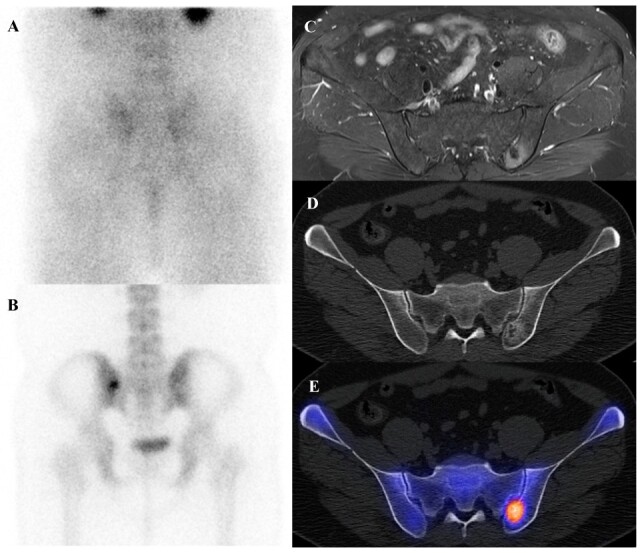
A 38-year-old male with osteoblastoma of the left iliac bone. (**A**,**B**) Bone scintigraphy (posterior view) shows no abnormal perfusion on the blood pool phase, but focal increased uptake in the left iliac bone on the delayed phase. (**C**) T2-weighted fat-suppressed magnetic resonance imaging demonstrates an irregular sclerotic lesion with surrounding bone marrow edema at the left iliac crest near the sacroiliac joint. (**D**,**E**) Bone single-photon emission tomography/computed tomography reveals an osteolytic lesion with a sclerotic rim and focal increased tracer uptake.

Osteoblastoma is a rare bone-forming tumor with a male predilection, most commonly occurring in the second to third decades of life. Its essential imaging features are a lytic lesion larger than 2 cm with well-defined borders and without permeation of the host bone. Osteoblastoma, a larger and more aggressive counterpart of osteoid osteoma, is histologically similar but shows greater growth potential with high vascularity and osteoblastic activity [66,67]. Consequently, they are intensely metabolically active and demonstrate markedly elevated [^18^F]FDG uptake on PET/CT [21,68]. Published reports document a wide range of high SUVmax in osteoblastomas, with averages around 3.2 in some series, individual cases reaching 6.2, and some spinal lesions exhibiting SUVmax as high as 16.0 [68,69,70,71]. This level of [^18^F]FDG avidity is well within the range of, and often exceeds that of, many malignant bone sarcomas. This significant overlap renders [^18^F]FDG PET incapable of reliably differentiating a benign osteoblastoma from a malignancy based on the intensity of uptake alone. The diagnosis relies on integrating the PET findings with the characteristic radiographic appearance and location. Figure 5 illustrates a case of osteoblastoma in the iliac bone, where bone scintigraphy, MRI, and SPECT/CT together demonstrated the characteristic features of this entity.

### 4.2. Cartilage-Forming Lesions

#### 4.2.1. Osteochondroma

**Figure 6:**
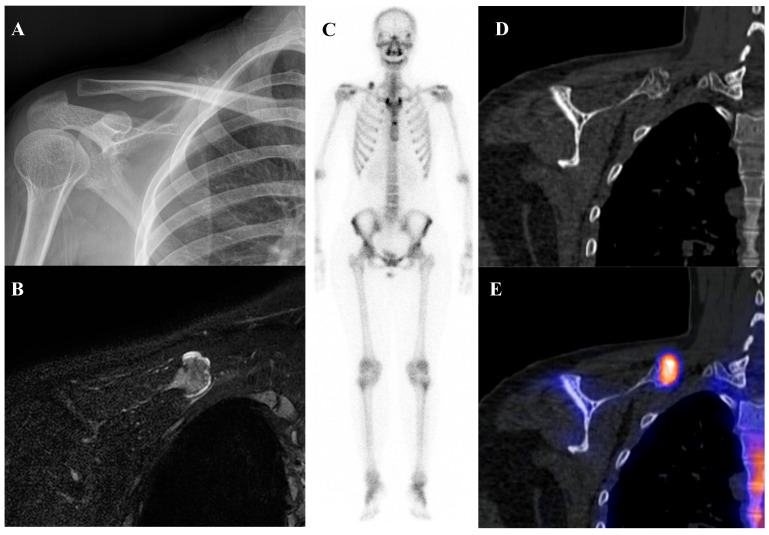
A 16-year-old female with osteochondroma of the right scapula. (**A**) Plain radiograph shows a protruding mixed sclerotic mass at the scapular cap. (**B**) T2-weighted fat-suppressed magnetic resonance imaging demonstrates a pedunculated, lobulated osseous lesion arising from the scapular cap, with continuity of the cortex and medulla and a thin, T2-bright enhancing cartilaginous cap. (**C**) Bone scintigraphy shows focal uptake at the lesion site. (**D**,**E**) Bone single-photon emission tomography/computed tomography demonstrates focal tracer uptake at the bone–cartilage junction.

Osteochondroma is the most common benign bone tumor, typically arising in the metaphyses of long bones and most frequently diagnosed in patients younger than 20 years [2]. On bone scintigraphy, the osseous portion of an osteochondroma accumulates tracer. Particularly intense tracer uptake is observed at the bone–cartilage junction of osteochondromas where active endochondral ossification is taking place [72]. The primary role of nuclear medicine is in the surveillance for malignant transformation to a secondary chondrosarcoma, a rare but serious complication. A sudden, marked, and heterogeneous increase in tracer uptake in a previously stable osteochondroma, particularly in an adult, is highly suspicious for malignant change [73]. [^18^F]FDG PET/CT is superior to bone scintigraphy for this purpose, as a significant increase in SUVmax provides a more specific and quantifiable marker of malignant transformation than a general increase in osteoblastic activity. Osteochondromas generally show no or faint [^18^F]FDG uptake, with approximately one-third of patients showing uptake higher than that of the background [54,64,74]. Figure 6 illustrates a typical case of scapular osteochondroma, in which MRI, bone scintigraphy, and SPECT/CT demonstrated the characteristic features of this lesion.

#### 4.2.2. Chondroblastoma

**Figure 7:**
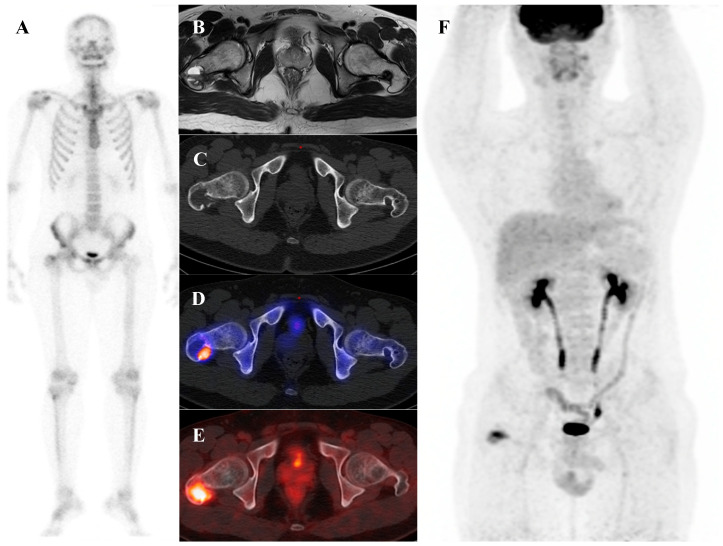
A 22-year-old male with chondroblastoma of the right femoral greater trochanter. (**A**) Bone scintigraphy shows focal uptake. (**B**) T2 fat-suppressed magnetic resonance imaging reveals an osteolytic mass with cortical disruption, extraosseous extension, fluid–fluid levels suggesting hemorrhage, nodular rim enhancement, and peritumoral edema, mimicking a malignant tumor with secondary aneurysmal bone cyst. (**C**,**D**) Bone single-photon emission tomography/computed tomography (CT) demonstrates a well-defined osteolytic lesion with a sclerotic rim and intense uptake at the site of extraosseous extension. (**E**,**F**) [^18^F]FDG positron emission tomography/CT shows intense uptake (SUVmax 9.8) at the lesion site. Surgical excision confirmed chondroblastoma with secondary aneurysmal bone cyst change.

Chondroblastoma is a rare, benign cartilaginous tumor that characteristically arises in the epiphysis of long bones in adolescents and young adults [3,75]. Similarly to osteoblastoma, chondroblastoma is a significant mimic of malignancy on functional imaging. It is a metabolically active tumor that demonstrates intense uptake on both bone scintigraphy and [^18^F]FDG PET/CT [76,77]. Figure 7 demonstrates such a case, showing a chondroblastoma of the femoral greater trochanter with cortical disruption, extraosseous extension, and increased uptake on both bone scintigraphy and [^18^F]FDG PET/CT. The key to differential diagnosis is context: the combination of its classic epiphyseal location in a skeletally immature or young adult patient, along with its characteristic radiographic appearance (a lytic lesion with a thin sclerotic rim), should strongly suggest chondroblastoma despite the high metabolic activity [78]. Although metastasis from chondroblastoma is exceedingly rare, the whole-body survey capability of bone scintigraphy and [^18^F]FDG PET/CT may still be considered valuable in comprehensive staging and in the rare detection of distant involvement [79,80,81].

#### 4.2.3. Enchondroma

**Figure 8:**
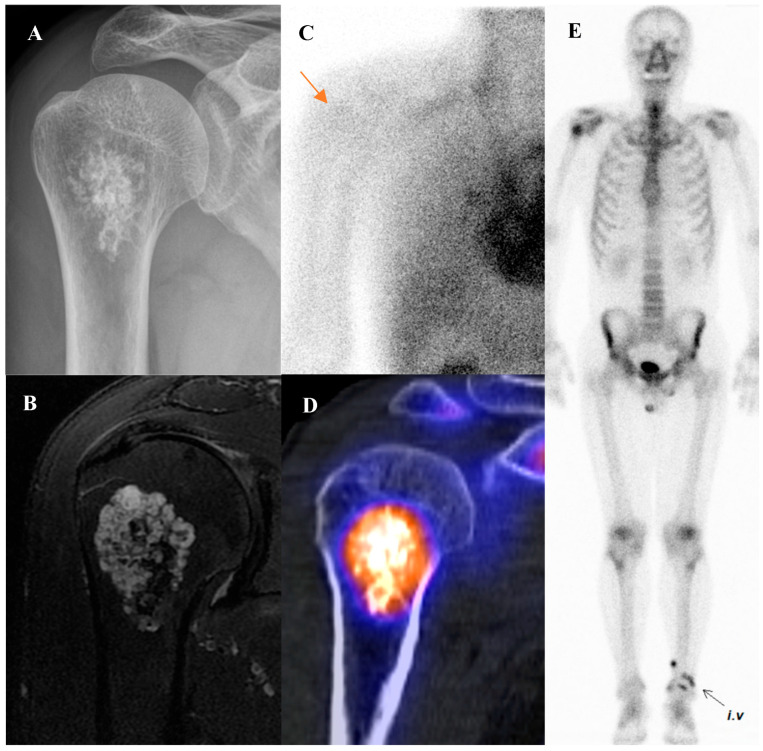
A 58-year-old male with enchondroma of the right humerus. (**A**) Plain radiograph shows popcorn-like calcifications at the humeral neck. (**B**) T2-weighted fat-suppressed magnetic resonance imaging demonstrates a lobulated intramedullary tumor in the proximal metaphysis, showing predominantly high signal intensity with focal low to intermediate signal areas. (**C**,**E**) Bone scintigraphy reveals mildly increased perfusion on the blood pool phase (arrow) and moderate uptake on the delayed phase at the lesion site. (**D**) Bone single-photon emission tomography/computed tomography demonstrates increased uptake at the proximal humeral metaphysis, corresponding to the calcified lesion.

Enchondroma is a common benign medullary cartilage tumor that typically arises within phalanges, the femur, and the humerus [54,82]. It occurs equally in men and women, with approximately 60% of cases presenting between the ages of 10 and 39 and a peak incidence in the third decade [75,82]. Most enchondromas are asymptomatic and discovered incidentally [73]. On nuclear medicine imaging, enchondromas demonstrate none to moderately increased uptake on bone scintigraphy, depending on the extent and degree of mineralization at different evolutionary stages [72]. On PET/CT, they typically show minimal to mild [^18^F]FDG uptake, reflecting their low metabolic activity [54]. Figure 8 demonstrates a representative case of enchondroma, showing characteristic calcifications, lobulated intramedullary morphology, and mild to moderate tracer uptake on bone scintigraphy and SPECT/CT.

The major diagnostic challenge is differentiating a benign enchondroma from a low-grade chondrosarcoma (atypical cartilaginous tumor) when imaging features overlap. Advanced functional imaging approaches are under investigation to improve this distinction, as highlighted in Section 3.3 and Section 5.

### 4.3. Cystic and Vascular Lesions

#### 4.3.1. Aneurysmal Bone Cyst (ABC)

**Figure 9:**
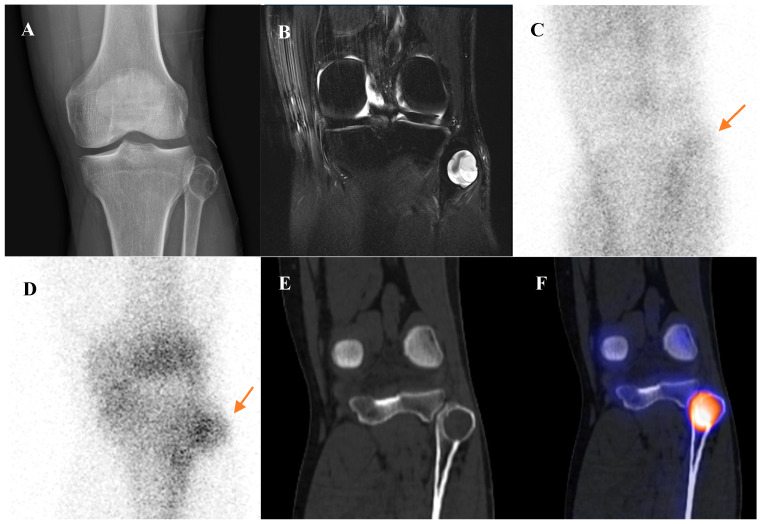
A 45-year-old male with an aneurysmal bone cyst (ABC) of the left proximal fibula. (**A**) Plain radiograph shows a sharply defined, expansile, lucent lesion of the proximal fibula. (**B**) T2-weighted fat-suppressed magnetic resonance imaging demonstrates a multiloculated cystic mass with preserved cortical continuity but bulging deformity. Fluid–fluid levels are present within the chambers, without solid enhancing components, and overlying soft-tissue swelling is noted. (**C**,**D**) Bone scintigraphy reveals mildly increased perfusion on the blood pool phase and focal uptake on the delayed phase (arrows). (**E**,**F**) Bone single-photon emission tomography/computed tomography demonstrates tracer uptake predominantly along the cystic lesion margin. The lesion was surgically excised and confirmed as ABC.

Aneurysmal bone cyst is an expansile, blood-filled, osteoclastic giant cell-rich tumor of bone. It is commonly seen in patients younger than 20 years and typically occurs in the long bones, spine, and pelvis [73,75,78,83,84]. The classic finding on bone scintigraphy is the “doughnut sign,” which consists of a ring of intense tracer uptake in the hypervascular fibrous septa and reactive bone at the periphery of the lesion, surrounding a central photopenic (cold) core that corresponds to the blood-filled cystic cavity [72]. Figure 9 demonstrates a representative case of an ABC in the proximal fibula, showing a multiloculated cystic mass with fluid–fluid levels on MRI, a sharply defined expansile lucency on radiograph, and peripheral tracer uptake on bone scintigraphy and SPECT/CT. While this sign is characteristic, it is not specific to ABC. It can also be seen in other lesions with central necrosis or cystic change, including giant cell tumor, chondroblastoma, and even telangiectatic osteosarcoma [72]. [^18^F]FDG uptake in an ABC is variable [85]. In primary ABCs, uptake is typically confined to the solid peripheral components. If the ABC is secondary to another underlying tumor or antecedent trauma, the [^18^F]FDG avidity will largely reflect the metabolic activity of that primary lesion [54,75,77].

#### 4.3.2. Simple (Unicameral) Bone Cyst (SBC)

**Figure 10:**
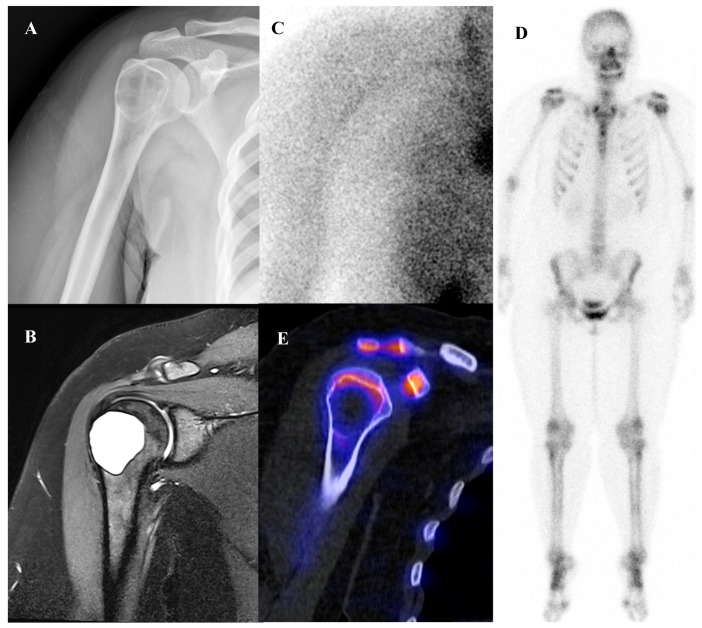
A 19-year-old female with a simple bone cyst of the right proximal humerus. (**A**) Plain radiograph demonstrates a well-defined lucent lesion. (**B**) T2-weighted fat-suppressed magnetic resonance imaging shows a lobulated, T2-bright cystic lesion at the metaphysis, adjacent to the physis, with thin rim enhancement and a T2 shine-through phenomenon. (**C**,**D**) Bone scintigraphy reveals no significant uptake in the right proximal humerus on either the blood pool or delayed phases. (**E**) Bone single-photon emission tomography/computed tomography similarly shows no significant tracer uptake at the lesion site. Growth plate shows mildly increased radiotracer uptake.

A simple bone cyst, or unicameral bone cyst, is a true fluid-filled cavity lined by a thin fibrous membrane [72,75]. In its typical, unfractured state, a SBC is an avascular lesion with minimally increased tracer uptake in the rim [72]. Consequently, it classically appears as a photopenic or “cold” lesion on all three phases of a bone scan. Figure 10 illustrates the characteristic imaging appearance of a simple bone cyst, with minimal metabolic activity on nuclear medicine studies, underscoring its distinction from other cystic bone lesions. However, this classic appearance is often altered by a pathologic fracture, which is the presenting symptom in the majority of cases. In such situations, the value of planar bone scintigraphy is limited, as uptake related to fracture may obscure the underlying lesion. Bone SPECT/CT can help overcome this limitation by localizing tracer uptake to the fracture site and delineating its relationship to the cyst, thereby improving diagnostic confidence.

The summary of nuclear medicine findings of benign bone tumors is presented in Table 1.

## 5. Future Directions

### 5.1. Quantitative SPECT (qSPECT) and CZT SPECT/CT

Recent advances in SPECT/CT technology have markedly improved image quality and enabled quantitative analysis. New gamma camera designs leverage solid-state cadmium zinc telluride (CZT) detectors and novel collimators, achieving higher sensitivity and spatial resolution than conventional Anger cameras [86,87,88]. These hardware innovations, coupled with resolution recovery iterative reconstruction algorithms, yield sharper images and allow faster scans with improved lesion detectability [87]. Modern SPECT software now incorporates system-specific calibration and corrections so that tracer uptake can be quantified in absolute terms. SPECT has thus evolved from purely qualitative interpretation to quantitative imaging, with voxel values expressed as activity concentration or SUVs [89].

Early studies show that quantitative SPECT/CT improves lesion characterization and reader agreement, with diagnostic accuracy for bone metastases often exceeding conventional qualitative reads [90]. As noted in Section 3.3, few studies have explored the diagnostic utility of SUV measurements in distinguishing benign from malignant primary bone tumors [57,91,92,93]. Although current evidence in bone tumors is limited to small, single-center cohorts, emphasizing the need for multicenter validation, these technological advances collectively bring SPECT/CT closer to PET/CT in terms of image quality and quantification, expanding its potential role in differential diagnosis, staging, and therapy monitoring.

### 5.2. Radiomics and Artificial Intelligence (AI)

Radiomics has emerged as a promising approach to capture intratumoral heterogeneity and improve risk stratification in bone tumors. Early [^18^F]FDG PET/CT-based studies demonstrated that texture-derived features can complement conventional SUV metrics, enhancing response prediction and prognostic assessment in sarcomas [94,95,96]. Radiomics applied to SPECT/CT has demonstrated potential in differentiating malignant lesions from benign bone tumors. In a study of long-bone cartilage tumors, Yoon et al. identified zone-length non-uniformity (ZLNUGLZLM) as an independent predictor of ACT, with reported sensitivities of 83–85% and specificities of 58–91% across validation cohorts [97]. These findings suggest that radiomic modeling of quantitative SPECT/CT data may provide incremental value over SUVmax alone, potentially improving the noninvasive discrimination between benign enchondroma and low-grade chondrosarcoma.

Beyond radiomics, artificial intelligence (AI) is increasingly being applied to musculoskeletal oncology. In a systematic review designed to evaluate the performance of AI techniques for differentiating benign from malignant bone lesions, Ong et al. reported that PET/CT-based studies achieved accuracies of 0.74–0.88, sensitivities of 0.84–0.90, and specificities of 0.74–0.85, with AUC values ranging from 0.76 to 0.95 [98]. In a large multicenter study of 880 patients, von Schacky et al. developed machine learning models based on radiomic features extracted from radiographs, combined with demographic information, to differentiate benign from malignant primary bone tumors [99]. Their best-performing artificial neural network achieved an AUC of 0.90 on an external validation set, with 75% accuracy, 90% sensitivity, and 68% specificity—performance that was lower than expert musculoskeletal radiologists but higher than radiology residents. These results demonstrate that AI-based radiographic models can support less experienced readers in improving diagnostic accuracy.

Despite these advances, current radiomics and AI studies remain limited by small sample sizes, retrospective designs, and lack of multicenter validation in nuclear medicine imaging. To date, no dedicated AI studies have specifically addressed nuclear medicine imaging of primary bone tumors, representing an important gap for future research. Standardization of acquisition protocols, harmonization of feature extraction, and collaborative multicenter efforts will be essential before these approaches can be reliably translated into clinical practice.

### 5.3. [^18^F]Sodium Fluoride (NAF) PET/CT

On the other hand, [^18^F]NaF serves as a well-known PET radiotracer that specifically targets bone metabolism. When introduced into the body, the [^18^F]fluoride ion exchanges with hydroxyl groups in hydroxyapatite crystals present on the surface of the bone matrix, leading to the formation of fluorapatite. The uptake of [^18^F]NaF thus reflects blood flow and osteoblastic activity, like [^99m^Tc]Tc-MDP. Unlike [^99m^Tc]Tc-MDP, however, [^18^F]NAF PET/CT benefits from the superior pharmacokinetics, higher spatial resolution, and quantitative capabilities of PET imaging [100]. Multiple studies and meta-analyses have shown that [^18^F]NaF PET/CT has significantly higher sensitivity and diagnostic accuracy than conventional planar bone scintigraphy and SPECT for the detection of osseous metastases [101].

[^18^F]NaF PET/CT provides highly sensitive assessment of bone turnover and complements [^18^F]FDG PET by detecting skeletal metastases and skip lesions. In osteosarcoma, it has revealed rare metastatic sites and led to the proposal of NAFCIST (NaF PET Response Criteria in Solid Tumors) for therapy monitoring in patients who underwent [^223^Ra]RaCl_2_ therapy [102]. In early studies, NAFCIST correlated with biomarkers and overall survival, whereas [^18^F]FDG-based PERCIST did not [103]. These findings suggest [^18^F]NaF PET may serve as a valuable staging and prognostic tool, though larger multicenter validation is needed.

### 5.4. [^68^Ga]Ga-Fibroblast Activation Protein Inhibitor (FAPI) PET/CT

Fibroblast activation protein (FAP) is a type II serine protease that is overexpressed on cancer-associated fibroblasts and mesenchymal tumor cells in the stroma of sarcomas [104]. By targeting this FAP-rich microenvironment, [^68^Ga]Ga-FAPI PET tracers yield exceptionally high tumor-to-background contrast and minimal physiologic uptake, providing superior lesion conspicuity within the musculoskeletal system compared with glucose-based imaging [105,106].

Several studies have demonstrated its complementary value to [^18^F]FDG PET/CT, particularly in low-grade or low [^18^F]FDG-avid sarcomas. Lanzafame et al. reported higher SUVmax with [^68^Ga]Ga-FAPI-46 than [^18^F]FDG (10.4 ± 8.5 vs. 7.0 ± 4.5, *p* = 0.01) [107]. Sakir et al. found improved detection of bone and hepatic metastases with [^68^Ga]Ga-FAPI PET imaging despite [^18^F]FDG identifying more total lesions [108].

[^68^Ga]Ga-FAPI PET/CT also shows promise as a theranostic tool, with early experience using [^90^Y]Y-FAPI-46 and [^177^Lu]Lu-FAPI-2286 demonstrating favorable safety and preliminary efficacy in advanced sarcomas [109,110,111]. However, variable FAP expression and uptake in inflammatory tissue remain limitations [104]. Larger multicenter trials are needed to validate its diagnostic and prognostic roles and to standardize quantitative parameters for sarcoma assessment.

### 5.5. Other Emerging Radiotracers

Several novel PET tracers are under investigation for musculoskeletal oncology. Prostate-specific membrane antigen–targeted PET/CT using [^68^Ga]Ga-PSMA-11, originally developed for prostate cancer, has occasionally demonstrated uptake in osteosarcomas, Ewing sarcomas, and other high-grade soft-tissue sarcomas, suggesting potential relevance for tumor characterization and theranostic applications [112].

[^68^Ga]Ga-DOTA-somatostatin analogues (e.g., DOTATATE, DOTATOC) have shown high sensitivity in detecting phosphaturic mesenchymal tumors responsible for tumor-induced osteomalacia (TIO), owing to their strong somatostatin-receptor expression [113]. These tumors often arise within bone or adjacent soft tissues and may be small and clinically elusive on conventional imaging. Somatostatin-receptor PET/CT enables precise localization of these lesions, facilitating surgical resection and complete biochemical cure.

## 6. Conclusions

Nuclear medicine imaging has become integral to the evaluation of primary bone tumors, complementing conventional radiological modalities by providing unique functional and molecular information. [^18^F]FDG PET/CT is now a cornerstone for guiding biopsy, tumor grading, staging, treatment response assessment, and recurrence evaluation of sarcomas, while SPECT/CT offers quantitative capabilities and radiomic features that may refine diagnostic accuracy, especially in cartilaginous tumors.

Nevertheless, several limitations should be acknowledged. The specificity of both bone scintigraphy and [^18^F]FDG PET/CT remains variable, as benign or inflammatory conditions may show increased uptake leading to potential false-positive findings [9,28]. In addition, the availability of advanced tracers and hybrid imaging systems is still limited in many institutions, restricting their broader clinical implementation.

These limitations have driven ongoing efforts to develop new molecular tracers with improved specificity and wider applicability. Emerging agents—such as [^68^Ga]Ga-FAPI, [^68^Ga]Ga-PSMA-11, and [^68^Ga]Ga-DOTA-somatostatin analogues—show promise for enhancing lesion characterization, detecting low-[^18^F]FDG-avid tumors, and expanding theragnostic opportunities in bone tumor imaging.

Overall, continued advances in quantitative imaging, radiomics, artificial intelligence, and novel tracer development are expected to further strengthen the role of nuclear medicine in bone tumor evaluation, paving the way for more precise, personalized, and biologically informed oncologic care.

## Figures and Tables

**Table 1:** Summary of Nuclear Medicine Characteristics of Benign Bone Tumors.

Benign Tumor	Typical Location /Age	Bone Scan	[^18^F]FDG PET/CT	Key Diagnostic Clues & Pitfalls
Osteoid Osteoma	Long bone diaphysis/cortex; 10–35 yrs	Intense focal uptake in all phases (“double-density sign”)	Usually high uptake in nidus	Highly sensitive for localization of occult lesions. Classic clinical history.
Osteoblastoma	Spine (posterior elements); <30 yrs	Intense uptake	Very high uptake mimics malignancy	A major diagnostic pitfall. High uptake is not specific for malignancy.
Osteochondroma	Metaphysis of long bones especially around the knee; <20 yrs	Mild–moderate uptake, similar to physis	Low uptake unless transformed	Sudden increase in uptake is suspicious for malignant transformation.
Enchondroma	Phalanges, femur, and humerus; 10–39 yrs (peak 3rd decade)	None–moderate uptake	Minimal–mild uptake	Difficulty in differentiating from low grade chondrosarcoma.
Chondroblastoma	Epiphysis of long bones; 10–30 yrs	Intense uptake	High uptake mimics malignancy	Classic epiphyseal location in a young patient is the key clue.
ABC	Metaphysis of long bones, spine, pelvis; <20 yrs	“Doughnut sign”	Variable, often peripheral uptake	Doughnut sign is not specific. MRI fluid-fluid levels are characteristic.
SBC	Metaphysis of proximal humerus/femur, abutting the growth plate; <20 yrs	Photopenic (“cold”) unless fractured	Photopenic (“cold”) unless fractured	Fracture causes peripheral uptake, mimicking ABC.

Abbreviations: yrs = years; CT = computed tomography; MRI = magnetic resonance imaging; PET = positron emission tomography; FDG = fluorodeoxyglucose; ABC = aneurysmal bone cyst; SBC = simple bone cyst.

## Data Availability

The dataset is available on request from the authors. The data are not publicly available due to institutional policy.
